# Double venous drainage through the superior vena cava in minimally invasive aortic valve replacement: a retrospective study

**DOI:** 10.3325/cmj.2012.53.11

**Published:** 2012-02

**Authors:** Tomislav Klokočovnik, Tanja Kersnik Levart, Matjaž Bunc

**Affiliations:** 1 Department of Cardiovascular Surgery, University Medical Centre Ljubljana, Ljubljana, Slovenia; 2 Department of Pediatric Nephrology, University Medical Centre Ljubljana, Ljubljana, Slovenia; 3 Department of Cardiology, University Medical Centre Ljubljana, Ljubljana, Slovenia

## Abstract

**Aim:**

To compare the outcomes of patients who underwent upper mini-sternotomy or right mini-thoracotomy and those who underwent full sternotomy and to report a technical improvement in venous drainage by means of double venous cannulation of the superior vena cava (SVC) in mini surgical procedures.

**Methods:**

We retrospectively analyzed the outcome of 217 patients who underwent aortic valve replacement through upper mini-sternotomy or right mini-thoracotomy at the Department of Cardiovascular Surgery, University Medical Centre Ljubljana, Slovenia from 1996 till 2010. Cannulation of SVC and right atrial appendage was performed in 142/217 (65%) patients, while in the remaining 75 (35%) patients, double cannulation of SVC was used for venous drainage. The results of patients who underwent mini approaches were compared to 236 patients who underwent full sternotomy for the same purpose from 2009 to 2010 at the same center.

**Results:**

We found a shorter mean length of intensive care unit stay, less volume chest-tube drainage, shorter crossclamp and cardio pulmonary bypass times, and less postoperative permanent pacemaker implantations in the minimally invasive group patients than in full sternotomy group patients. Using double cannulation of the SVC for venous drainage made venous cannulation in mini approaches easier and eliminated the need for obtaining femoral venous access.

**Conclusion:**

Our study confirmed that even though technically challenging, upper mini-sternotomy and right mini-thoracotomy approaches for aortic valve replacement have potential advantages over conventional median sternotomy. They were proved to be safe, efficacious, and can significantly reduce surgical trauma and are therefore particularly valuable in some higher risk, obese, diabetic and elderly patients. Using double cannulation of SVC for venous drainage made venous cannulation easier and eliminated the need for obtaining femoral venous access.

Minimally invasive cardiac valve surgery for patients with isolated valve pathology was introduced at the Department of Cardiovascular Surgery, University Medical Centre Ljubljana, Slovenia in 1996, the same year as at the Brigham and Women’s Hospital Boston, Loma Linda University, and the Cleveland Clinic (1,2). Less invasive approaches (upper mini-sternotomy, right mini-thoracotomy) confer many advantages when compared to median sternotomy, which is still considered a standard approach for the surgical repair or replacement of cardiac valves (3). It is true that the latter offers excellent exposure of the operating field, however the less invasive approaches lead to a smaller surgical wound and potentially less blood loss, decreased risk of infection, shorter intubation period, decreased postoperative pain, earlier hospital discharge, and a smaller, cosmetically more acceptable post-operative scar (4,5). Moreover, when re-operation is needed after mini incisions, it is less hazardous because the pericardium has not been completely dissected (6). They, on the other hand, can be quite challenging for the surgeon, sometimes making the standard venous cannulation of the superior vena cava (SVC) and right atrial appendage impossible due to insufficient exposure of the right atrial appendage and therefore constraining the surgeon to other ways of venous drainage. One of such ways is the well known cannulation of the SVC and femoral vein, where patient is subjected to femoral venous access (7). In order to be as little invasive as possible and avoid the latter, we performed a double venous cannulation of the SVC, first in the patients in whom a standard venous drainage was not possible and then also as a primary means of venous drainage.

The aim of this study was to present the outcome of 217 patients who underwent aortic valve replacement through upper mini-sternotomy or right mini-thoracotomy and compare it to our contemporary full sternotomy cohort and to report our new technical improvement in venous drainage by means of double venous cannulation of the SVC in mini surgical procedures.

## Patients and methods

We retrospectively analyzed the outcome of 217 patients who underwent aortic valve replacement through an upper mini-sternotomy (n = 209) or right mini-thoracotomy (n = 8) at the Department of Cardiovascular Surgery, University Medical Centre Ljubljana, Slovenia since 1996. These results were compared to 236 patients who underwent full sternotomy for the same purpose from 2009 to 2010 at the same center. It should be noted that minimally invasive cardiac valve procedures were all done by the same surgeon, while full sternotomy procedures were performed by several surgeons. Patients underwent one of the two mini approaches when they had an isolated aortic valve disease and when they were appointed to the surgeon who routinely performs these procedures in our center. The decision which mini approach to use was entirely that of the surgeon. Obesity, diabetes mellitus, and chronic obstructive pulmonary disease were not regarded contraindications for these mini approaches; however patients with coronary artery disease or aneurysm of the ascending aorta were not operated on in this way. Five of our patients had undergone prior valve replacement, which in our judgment was not a contraindication for mini approach surgery. Other 236 patients underwent standard full sternotomy. The most common etiology of valvular disease in both groups of patients was calcific aortic valve stenosis. Both groups of patients had very similar clinical characteristics (Table 1).

**Table 1:** Clinical characteristics of Slovenian patients who underwent mini surgical procedures and those who underwent median sternotomy

	No. (%) of patients with				
	right mini-thoracotomy	upper mini-sternotomy	both mini procedures	median sternotomy	*P*
Number of patients	8	209	217	236	
Age, years (median and range)	74 (64-83)	72 (33-78)	73 (33- 83)	69 (18-89)	0.10
Male:female	6:2	138:71	146:73	112:124	
Mean ejection fraction (%)	52	56	54	54	
Aortic stenosis	8	209	217	236	
NYHA class*					
I	0 (0)	0 (0)	0 (0)	53 (22)	
II	6 (75)	160 (76)	166 (76)	89 (38)	
III	2 (25)	49 (24)	51 (23)	79 (33)	
IV	0 (0)	0 (0)	0 (0)	15 (7)	
Cerebrovascular disease	1 (12)	27 (13)	28 (13)	30 (13)	
Lung disease	0 (0)	10 (5)	10 (5)	16 (6)	0.70
Peripheral vascular disease	1 (12)	17 (8)	18 (8)	19 (8)	
Renal disease	0 (0)	12 (6)	12 (5)	10 (4)	0.70
Atrial fibrillation	1 (12)	31 (15)	32 (14)	14 (6)	0.001
Coronary disease	2 (24)	37 (18)	39 (18)	35 (15)	0.50

*NYHA – New York Heart Association Functional Class.

Preoperatively, the hemodynamic significance of valvular damage was evaluated by means of noninvasive thransthoracic echocardiography and invasive angiography. After routine general anesthesia induction, a transesophageal echocardiography probe and external defibrillator pads were placed in all patients. In right mini-thoracotomy approach, 5-7-cm skin incision was made at the level of the third intercostal space, while at upper mini-sternotomy approach this incision was made along the sternum from the xyphoid to the level of the third intercostal space. In patients who underwent right mini-thoracotomy, the right internal mammary artery (RIMA) was sacrificed and the third costal cartilages were divided from the sternum. In both approaches, the pericardium was opened longitudinally and suspended to the skin to help expose the ascending aorta, right ventricular outflow tract, right atrium, and SVC. The aorta was retracted and mobilized with a tape to facilitate the exposure of the aortic root. Shortly after the patients had been fully heparinized, venous drainage was achieved by standard vacuum-assisted (40-60 mm Hg) venous cannulation of the SVC and right atrial appendage or vacuum-assisted (40-60 mm Hg) double venous cannulation of the SVC (22 French cannula, Medtronic, Inc., Minneapolis, MN, USA). Standard venous cannulation of the SVC and right atrial appendage was done in 142 patients, while double cannulation of the SVC was used for venous drainage in the remaining 75 patients. Arterial return was established by right femoral artery (in right mini-thoracotomy approach) or ascending aorta (in upper mini-sternotomy approach) (standard arterial cannula; Medtronic, Inc.). We cross-clamped the aorta and infused antegrade cold blood cardioplegia into the ascending aorta and retrograde cold blood cardioplegia into the coronary sinus to achieve cardiac arrest. The aortic valve was approached through a transverse aortotomy. The surgical field was flooded with carbon dioxide at a flow rate of 2.0 L/min. During aortic valve replacement, a vent was placed into the left ventricle through the aortic annulus. The native valve was removed completely and replaced with biological (porcine bioprosthesis Medtronic – mosaic ultra, stented aortic valve; Minneapolis, MN, USA) or mechanical (St. Jude – Medical, St. Paul, MN, USA) valve, secured by Teflon pledgeted sutures. Transesophageal echocardiography was used to monitor deairing of the heart and prosthetic valve function. Self-adhesive external defibrillator pads were used for defibrillation when necessary. After valve replacement and restoration of cardiac function, we deaired the heart through the aortic vent, followed by weaning from cardio pulmonary bypass (CPB), removal of the cannulae, and infusion of protamine. Electrodes that were placed on the right ventricular outflow tract and right atrium were used for temporary electrical stimulation. A drainage tube was inserted into the anterior mediastinum, and when necessary also into the right pleural area. Hemostasis was achieved and the wound was closed in layers. Before discharge, all patients underwent transthoracic echocardiography to monitor the function of the prosthetic valve.

The outcome of patients who underwent minimally invasive and those who underwent full sternotomy approach for aortic valve replacement were compared by χ^2^ test. *P* value <0.05 was considered significant.

## Results

Patients who underwent upper mini-sternotomy or right mini-thoracotomy and those who underwent full sternotomy had very similar clinical characteristics, with an exception that significantly more patients who underwent minimally invasive approaches had atrial fibrillation (Table 1). Minimally invasive group had significantly lower mean volume chest-tube drainage and significantly shorter mean crossclamp and CPB times, and mean length of ICU stay than full sternotomy group. Full sternotomy group had more postoperative permanent pacemaker implantations (Table 2, Table 3).

**Table 2:** Characteristics of different types of operation

	Right mini-thoracotomy	Upper mini-sternotomy	Both mini	Median sternotomy	*P*
Number	8	209	217	236	
Median crossclamp time in minutes (median, range)	58 (48-68)	54 (46-62)	56 (46-68)	87 (26-305)	0,001
Median cardio pulmonary bypass time in minutes (median, range)	82 (68-96)	78 (66-90)	80 (66-96)	119 (43-347)	0,001
Mean volume chest – tube drainage (cc)	235	385	310	638	0.001

**Table 3:** Postoperative outcomes of patients who underwent mini surgical procedures and those who underwent median sternotomy

	No. (%) of patients with				
	right mini-thoracotomy	upper mini-sternotomy	both mini	median sternotomy	*P*
Number	8	209	217	236	
30-d mortality	0	4 (2)	4 (1.8)	3 (1.2)	0.50
New atrial fibrillation	1 (12)	17 (9)	18 (8)	25 (11)	0.30
Conversion to sternotomy	0 (0)	5 (2)	5 (2)		
New permanent pacemaker	0 (0)	7 (3)	7 (3)	19 (8)	0.05
Stroke	0 (0)	2 (1)	2 (0.9)	4 (1.7)	0.50
Deep sternal wound infection	0 (0)	2 (1)	2 (0.9)	1 (0.4)	0.50
Reoperation for bleeding	1 (12)	12 (6)	13 (6)	22 (9)	0.30
Mean length of intensive care unit stay (hours)	26	28	27	136	0.001
Prolonged ventilation (>48 h)	0 (0)	43 (20)	43 (20)	64 (27)	0.20
Renal failure	0 (0)	14 (7)	14 (6)	21 (9)	0.30

**Figure 1:**
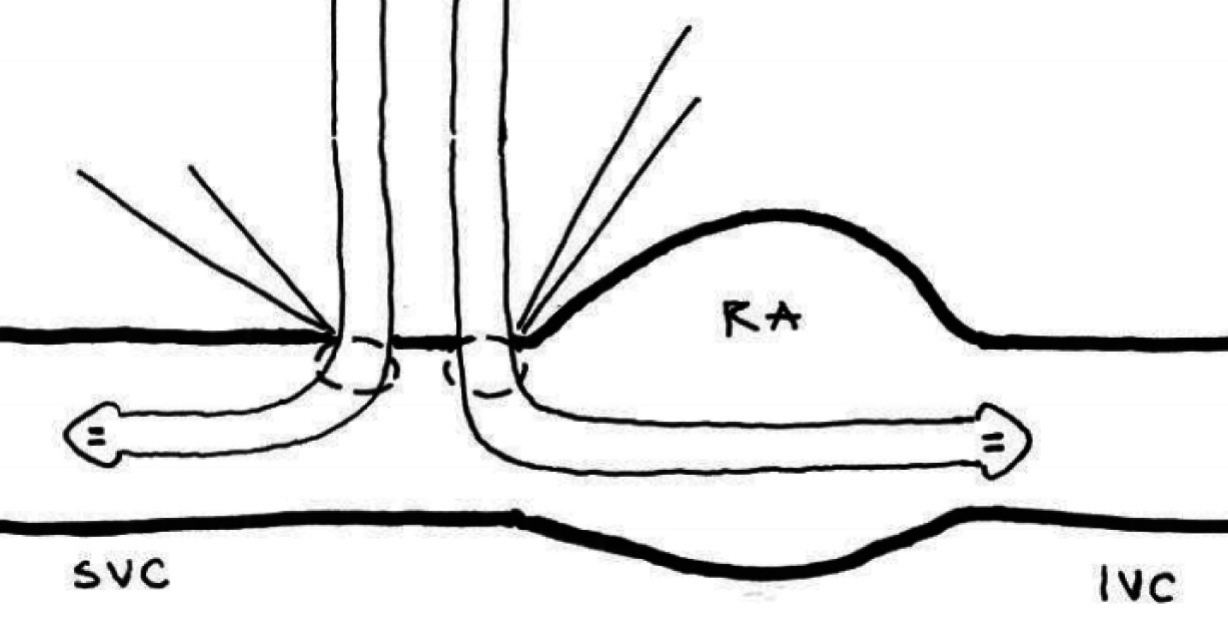
Venous drainage by double cannulation of superior vena cava. SVC – superior vena cava, IVC – inferior vena cava, RA – right atrium.

**Figure 2:**
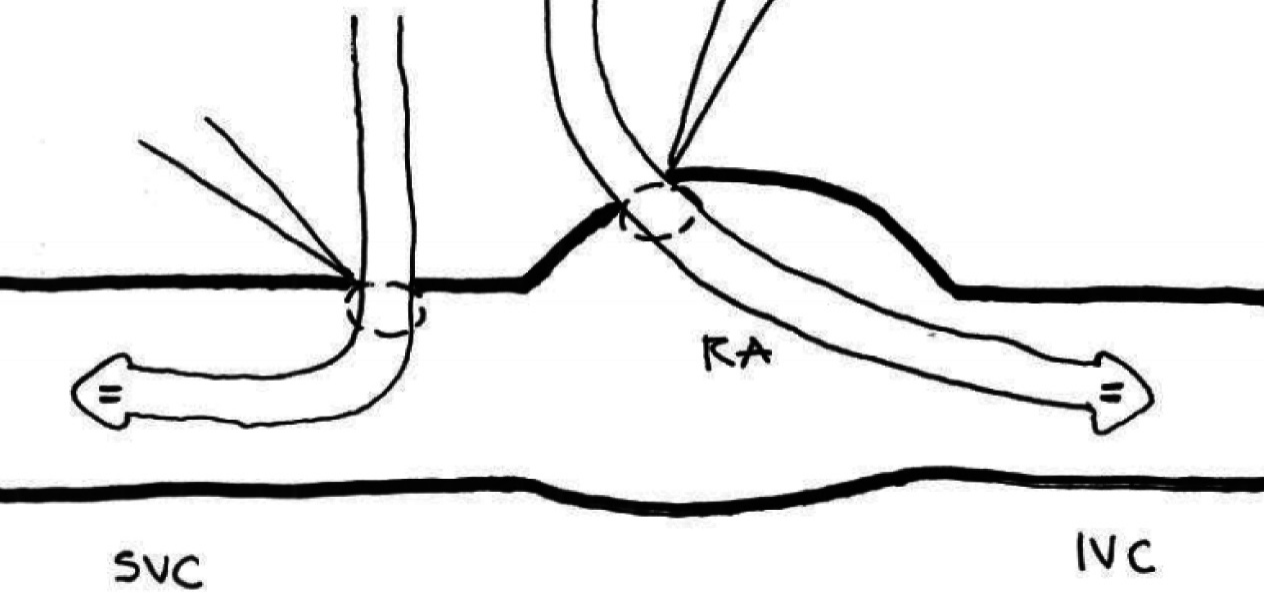
Standard venous drainage by cannulation of superior vena cava and right atrial appendage. SVC – superior vena cava, IVC – inferior vena cava, RA – right atrium.

**Figure 3:**
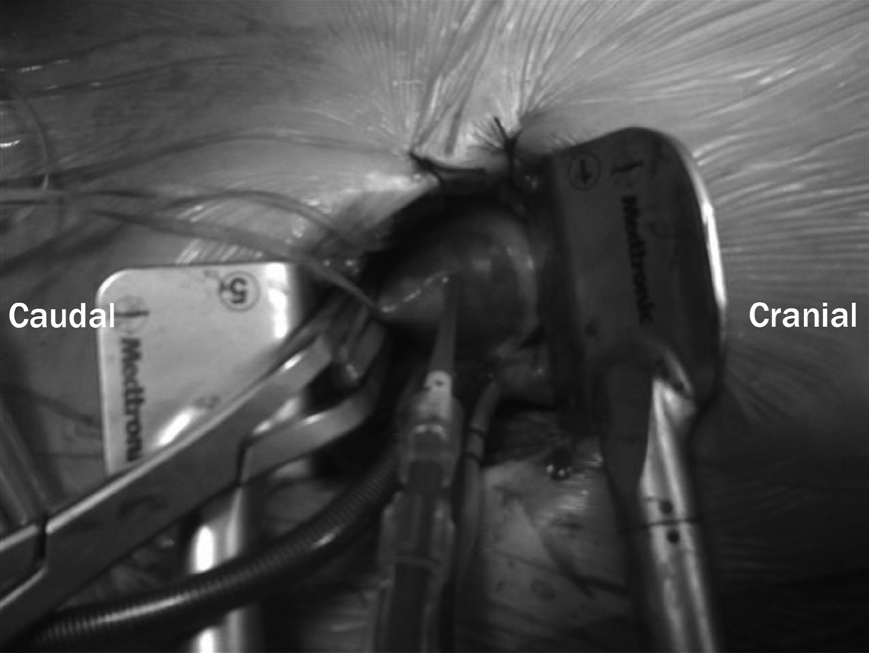
Surgical field obtained by right mini-thoracotomy with ascending aorta and antegrade blood cardioplegia.

**Figure 4:**
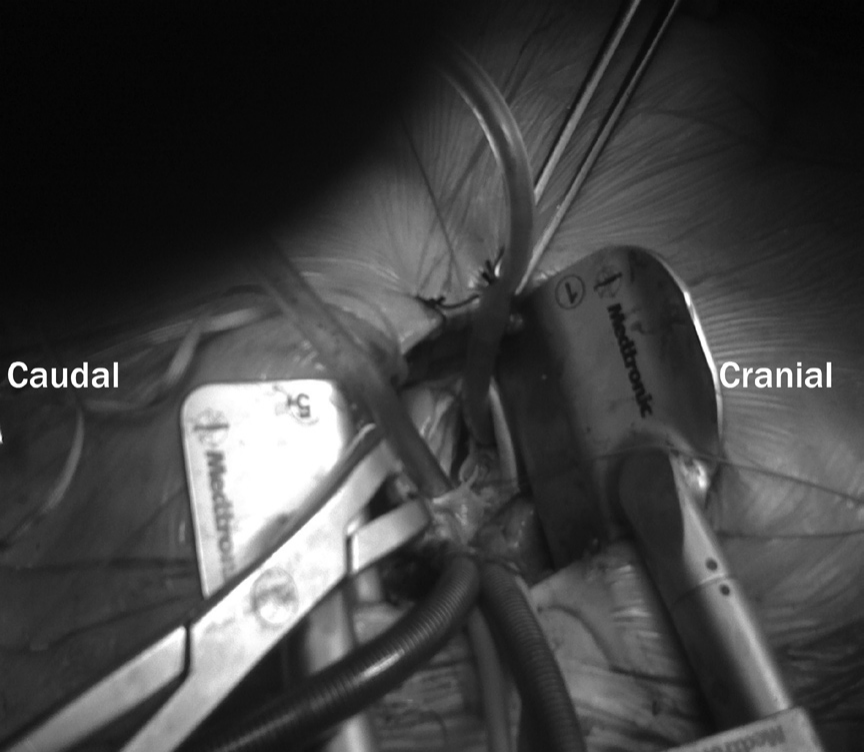
Surgical field obtained by right mini-thoracotomy with the aorta open and double venous cannulation of superior vena cava.

We also report what we believe is a technical improvement in venous drainage by means of double venous cannulation through the SVC (Figure 1-4). In this way, the need for obtaining femoral venous access was avoided in patients in whom it was not possible to perform standard cannulation of the SVC and right atrial appendage (Figure 2) due to insufficient intraoperative exposure of the right atrial appendage. Double venous cannulation of the SVC was performed in 75/217 (35%) patients. In 36 of them it was performed as a substitute for cannulation of the SVC and femoral vein when intraoperative exposure of the right atrial appendage was insufficient. In addition, it was used as a primary means of venous drainage in another 39 patients, making it all together 75 patients. In all of them, surgical fields obtained by upper mini-sternotomy and right mini-thoracotomy were sufficient and aortic valves were replaced without difficulty (Figure 3, Figure 4).

## Discussion

Our cohort of patients confirmed that less invasive approaches for aortic valve replacement confered many advantages and were superior in many ways to the conventional median sternotomy, which entails long incision through the skin and sternum and causes significant surgical trauma (4,5). We confirmed that mini approaches resulted in smaller surgical wound and potentially less blood loss, shorter intubation period, earlier hospital discharge, and a smaller, cosmetically more acceptable post-operative scar. It should be noted that our patients who underwent one of the two mini approaches had notably shorter median crossclamp times than shown in the literature (6,7). In addition, our patients who underwent standard median sternotomy had comparable CPB times to those from literature (6,7). On the other hand, our median crossclamp and CPB times in full sternotomy group were much longer than those in other studies and those in our minimally invasive procedure group, which could be explained by the fact that our full sternotomy procedures were performed by several surgeons, including those with only little experience, while minimally invasive procedures were all done by the same, well-trained surgeon.

Upper mini-sternotomy and right mini-thoracotomy can be performed with standard equipment and instruments and are potentially compatible with new technologies such as suture-less aortic valve prosthesis, which could be easily performed this way with a very short CPB time. The upper mini-sternotomy and right mini-thoracotomy approaches have several potential advantages over conventional sternotomy, however it should be noted that right mini-thoracotomy leads to loss of RIMA, violation of the right pleural space, and lung herniation, and that upper mini-sternotomy causes partial transection of the bone and potential instability of the sternum (8,9). In addition, minimally invasive approaches are sometimes technically harder to perform with limited operative field exposure. An exposure of the right atrial appendage can be insufficient, thus enabling standard venous drainage by means of venous cannulation of the SVC and right atrial appendage (1,10,11). In such cases, the surgeon is constrained to other ways of venous drainage. Cannulation of the SVC and femoral vein requires obtaining femoral venous access in addition to the incision at the level of the third intercostal space that is needed for the minimally invasive surgical approach (1,10,11). To the best of our knowledge, there is no report that describes a similar technical improvement in venous drainage that is performed in such cases without groin incision and that would use double venous cannulation of the SVC. In 75 (35%) our patients, this technique was used as a substitute for cannulation of the SVC and femoral vein when intraoperative exposure of the right atrial appendage was insufficient or as a primary means of venous drainage. All 75 of our patients were therefore spared the need for obtaining femoral venous access, while aortic valves were replaced without difficulty.

Taking all these advantages into consideration, together with the fact that obese patients and those with diabetes mellitus are prone to wound infection and are more liable to chest wall instability and respiratory distress, we believe that mini approaches should be considered for surgical repair or replacement of cardiac valves in patients at high risk of sternal dehiscence and respiratory complication (1,3,4,10-13). On the other hand, median sternotomy provides more access to the aortic root if the patient needs an aortic root procedure or has extensive atherosclerotic ascending aorta or arch.

Our study confirmed that even though technically challenging, upper mini-sternotomy and right mini-thoracotomy approaches for aortic valve replacement have potential advantages over conventional median sternotomy. They can offer easier exposure of the ascending aorta and effective aortic valve replacement especially when using double drainage of the SVC and when patients are reasonably selected for these procedures. These approaches have been proven to be safe and efficacious, very well tolerated, and cosmetically much more acceptable than conventional median sternotomy, especially when avoiding the need for obtaining femoral venous access by using double drainage of the SVC. Further studies are needed to refine the selection of the patients who would benefit from and be suitable for this approach and to determine the reproducibility between surgeons and institutions.
